# Digital and AI-Empowered Health Elements: An Integrated Pathway to Advancing Health

**DOI:** 10.34133/hds.0468

**Published:** 2026-05-15

**Authors:** Pengfei Li, Ying Zhou, Yan Li, Erdan Dong, Luxia Zhang

**Affiliations:** ^1^ National Institute of Health Data Science at Peking University, Beijing, China.; ^2^ Advanced Institute of Information Technology, Peking University, Hangzhou, Zhejiang Province, China.; ^3^ School of Nursing, Peking University, Beijing, China.; ^4^ Center for Smart and Healthy Buildings, Huazhong University of Science and Technology, Wuhan, China.; ^5^ School of Public Health, Shanghai Jiao Tong University School of Medicine, Shanghai, China.; ^6^ Department of Population Health Science and Policy, Icahn School of Medicine at Mount Sinai, New York, NY, USA.; ^7^ Department of Cardiology and Institute of Vascular Medicine, Peking University Third Hospital, Beijing, China.; ^8^ State Key Laboratory of Vascular Homeostasis and Remodeling, Peking University, Beijing, China.; ^9^ The Institute of Cardiovascular Sciences, Peking University, Beijing, China.; ^10^ Center for Digital Health and Artificial Intelligence, Peking University First Hospital ; Beijing Key Laboratory of Research, Development and Translational Application of Multimodal Intelligent Diagnosis and Treatment System, Beijing 100034, China.

## Abstract

Health outcomes arise from complex and evolving interactions among biological, behavioral, social, environmental, and technological influences. The social determinants of health framework have played a central role in establishing the importance of nonmedical drivers of health and continues to inform population health research and policy. As health data ecosystems expand and digital technologies become increasingly embedded in daily life, there is growing interest in approaches that can more explicitly represent dynamic, multidomain interactions shaping health in contemporary societies. In this Analysis, we introduce the concept of Health Elements, a framework that conceptualizes health as an emergent property distinct from complex additive effects arising from the interplay of biological, behavioral, social, environmental, and technological domains. We discuss how recent advances in data science and artificial intelligence create new opportunities to study these interactions, while highlighting the need for conceptual clarity, methodological rigor, and strong governance. We describe how Health Elements differ from and complement existing determinant-based models, outline principles for empirical operationalization, and consider implications for public health, clinical care, and policy. We also examine ethical, equity, and governance considerations relevant to integrated, artificial intelligence-enabled health systems. Finally, we identify key scientific questions to guide future research on when and how Health Elements approaches can advance health understanding and decision-making in an increasingly digital world.

## Introduction

Health is more than the absence of disease. It is a state of complete physical, mental, and social well-being sustained through interactions among biological, social, and environmental influences [1]. Although articulated more than 7 decades ago, this definition remains foundational. However, the contexts in which health is produced and maintained have changed substantially. Contemporary health systems operate within societies shaped by population aging, the growing burden of chronic noncommunicable diseases, environmental and climatic pressures, global mobility, and persistent social and health inequities [2–4]. These forces intersect across individual, community, and societal levels, generating complex patterns of risk and resilience.

The COVID-19 pandemic illustrated this complexity with particular clarity. While driven by a novel pathogen, its population-level impacts reflected interactions between biological susceptibility and social vulnerability, occupational exposure, housing conditions, mobility patterns, institutional trust, and digital access to information and services [5]. These influences shaped transmission dynamics, disease severity, and mortality across populations, reinforcing a long-standing public health insight: health outcomes arise from interacting systems rather than isolated causal pathways.

Despite this recognition, many dominant models in health research and practice continue to emphasize single-domain risk factors or downstream clinical events. Such approaches remain indispensable for biomedical discovery and clinical care, but they are limited in their ability to represent nonlinear dynamics, feedback processes, and dependencies across the life course [6]. Health reflects the cumulative effects of ongoing interactions between individuals and their biological characteristics, behaviors, social relationships, physical environments, and, increasingly, digital systems that shape access to care, exposure to information, and decision-making patterns.

Digital transformation has further reconfigured the health landscape. Algorithmic systems influence health-related behaviors and beliefs, digital platforms mediate access to services, and ubiquitous sensing technologies generate continuous streams of health-relevant data [7–9]. In parallel, advances in data science and artificial intelligence (AI) have enabled the integration of electronic health records, multiomics data, wearable sensors, environmental monitoring systems, and administrative datasets to represent health across the life course [7–11]. Machine learning methods capable of modeling high-dimensional and nonlinear relationships offer new opportunities to examine how diverse influences combine and evolve over time while also raising important conceptual, ethical, and governance questions [10,11].

In this context, we propose the concept of Health Elements. Health Elements conceptualize health as an emergent outcome of interacting biological, behavioral, social, environmental, and technological domains. Rather than replacing determinant-based approaches, the framework extends them by explicitly emphasizing interaction, temporality, and the growing role of digital systems in shaping health. Health Elements provide a structured orientation for investigating contemporary health complexity while remaining compatible with empirical rigor, ethical governance, and policy relevance.

## Conceptualizing Health Elements in the Digital Era

The social determinants of health (SDH) framework have been central to demonstrating that social and economic conditions are fundamental drivers of population health and health inequities [12]. Education, income, employment, housing, and social policy shape exposure to risk and access to resources across the life course, often exerting a stronger influence on health outcomes than medical care alone. By shifting attention away from purely biomedical explanations, SDH frameworks have profoundly reshaped public health research and policy.

However, SDH frameworks were largely developed in an era characterized by low-frequency data collection, limited linkage across domains, and minimal digital mediation of daily life. Determinants were therefore often conceptualized as relatively stable background conditions rather than as dynamic processes. While this conceptual framing was analytically useful, it offers limited guidance for modeling how determinants interact, how their effects evolve over time, or how emerging digital systems reshape health opportunities and risks.

The Health Elements framework builds on the conceptual foundations of SDH but introduces a process-oriented perspective. Rather than asking whether a given factor influences health, the framework emphasizes how multiple elements interact, under what conditions, and across which time horizons. This shift builds explicitly upon systems epidemiology and complexity science, which established over a decade ago that health outcomes arise from nonlinear interactions, feedback loops, and path-dependent trajectories and that complex systems dynamic models are essential tools for studying emergent population health patterns [6,13]. The Health Elements framework applies these principles to a contemporary, digitally mediated health landscape in which the configuration of relevant domains has expanded substantially. While the domains themselves have precedents across existing frameworks [12,14], the Health Elements framework’s distinguishing contribution lies not in its taxonomy but in its process-oriented architecture: the systematic study of how domains interact, under what conditions, and across which time horizons.

A defining extension of the Health Elements framework is the explicit inclusion of technological elements as constitutive components of health systems—a position that builds on a growing body of prior research [15–17]. The Health Elements framework advances these contributions by providing a scientific architecture within which the interactions, feedback, and temporal dynamics of technological elements alongside biological, behavioral, social, and environmental domains can be systematically studied. Digital infrastructures, data ecosystems, algorithmic decision systems, and platform-mediated interactions increasingly shape how health information is produced, interpreted, and acted upon [8,18]. These systems influence behavioral norms, access to care, allocation of resources, and the distribution of power within health systems. While existing integrative frameworks—including ecological and social determinants models—could incorporate digital or technological factors as cross-cutting modifiers, they typically subsume them under social, environmental, or system-level conditions. We argue that technological elements now warrant recognition as a structurally independent first-order domain for 3 reasons. First, technological systems introduce genuinely novel mechanisms of health influence—including algorithmic decision-making, platform-mediated behavioral environments, AI-enabled diagnostics, and real-time data feedback loops—that have no functional analog in predigital frameworks. Second, technological elements possess a uniquely cross-domain modifying capacity: They do not merely add an additional exposure, but actively restructure how biological, behavioral, social, and environmental elements operate, interact, and scale. Third, technological systems occupy a reflexive position in modern health systems—functioning both as determinants of health outcomes and as the primary infrastructure through which other determinants are measured, interpreted, and acted upon. Importantly, technological elements are not synonymous with advanced digital infrastructure. In low- and middle-income countries and other resource-constrained settings, the technological domain may be defined as much by absence or intermittency as by presence. Limited surveillance capacity, lack of interoperable health records, and low digital health literacy are themselves constitutive features of the technological environment that shape health outcomes and constrain disease detection and response.

In 2025, the Digital Health Institute for Transformation published a Periodic Table of Health Elements, a practitioner-oriented taxonomy cataloging 87 specific health factors across 5 weighted categories—Medical Care, Environment, Individual Behavior, Genetics and Biology, and Socioeconomic Circumstances—designed as an action roadmap for clinicians, policymakers, and innovators [19]. In that framework, digital and technological factors appear as subelements distributed within other categories, such as Telehealth Access within Medical Care and Digital Literacy within Socioeconomic Circumstances. The Health Elements framework differs in treating the technological domain as a structurally independent first-order constituent of health, reflecting the argument that digital infrastructure has distinct causal pathways and the capacity to amplify or attenuate the effects of all other domains. The 2 frameworks are complementary rather than competing: Digital Health Institute for Transformation provides a taxonomy and action roadmap for practice, while Health Elements provides a scientific architecture for investigating how domain interactions, feedback loops, and temporal dynamics produce emergent health outcomes.

Within the Health Elements framework, biological elements include physiology, genetics, epigenetics, molecular and cellular pathways, immune function, microbiome composition, and biological aging processes [20–23]. Behavioral elements include physical activity, diet, sleep, substance use, stress-related behaviors, and health literacy, many of which can now be observed through digital devices and continuous monitoring technologies [24,25]. Social elements arise from interpersonal relationships, socioeconomic conditions, education, cultural norms, and institutional contexts [12,26]. Environmental elements encompass natural and built environments, including air and water quality, climate exposures, housing conditions, transportation systems, and urban form [27–29]. Technological elements capture digital access, data availability and interoperability, algorithmic systems, and digital literacy, all of which shape how individuals and populations engage with health systems [30–32].

**Fig. 1.**
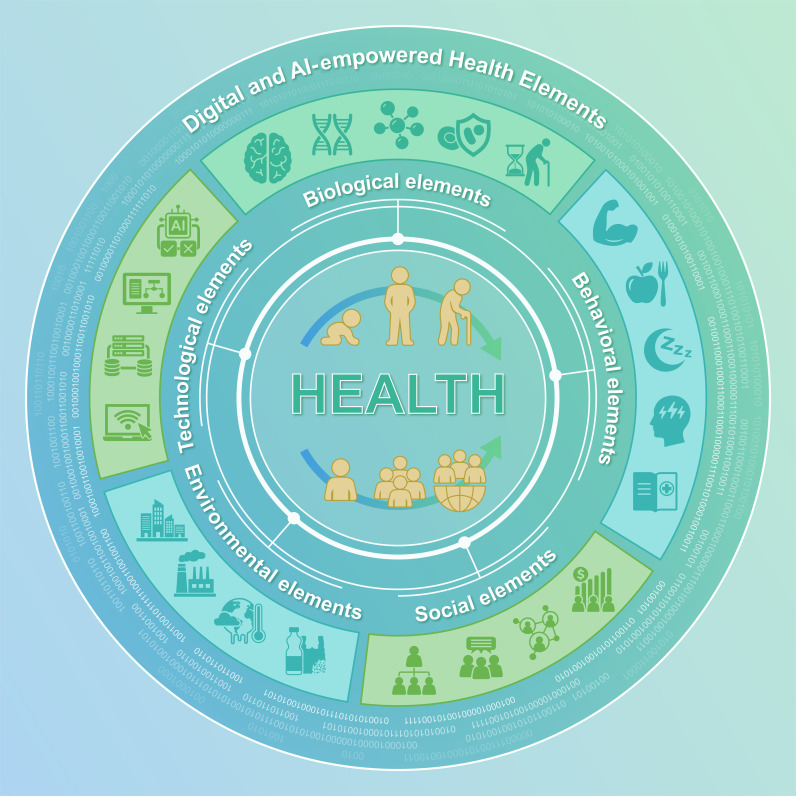
Conceptual framework of Health Elements in a digitally mediated health system. This figure illustrates the Health Elements framework, in which health is conceptualized as the result of interacting biological, behavioral, social, environmental, and technological domains. These elements operate across the life course and interact at multiple levels, from individuals to communities and populations. Digital data infrastructures and artificial intelligence support the integration and analysis of information across domains.

Health is conceptualized not as the additive sum of these elements, but as an emergent property of their configurations. In complexity science, emergence refers to system-level outcomes that arise from interactions among components and that cannot be predicted from—or reduced to—the properties of any individual component, even when those properties are fully specified [33]. This differs fundamentally from complex additive effects, in which multiple domains contribute independently to an outcome that is, in principle, decomposable into its constituent parts. Genuine emergence is characterized by nonlinearity, context dependence, and feedback dynamics: identical biological risk factors may produce different health trajectories depending on the configuration of social, environmental, behavioral, and technological elements operating simultaneously and over time. The shifting epidemiology of chronic kidney disease (CKD) in China illustrates this distinction: the rise of diabetes as the leading cause of CKD cannot be attributed to biological change alone but reflects the convergence of urbanization, behavioral transition, environmental exposure, and health system capacity—a pattern that no single-domain model could have anticipated or explained (Box 1). This relational perspective is central to the Health Elements approach and distinguishes it from frameworks that treat determinants as independent contributors (Fig. 1).

Box 1. A schematic Health Elements analysis of chronic kidney disease (CKD)

**Table:** 

**Example 1: The shifting epidemiology of CKD in China** A national surveillance study revealed that diabetes surpassed glomerular disease to become the leading cause of CKD in China since 2011[45]—a transition that cannot be explained by biological factors alone. Within a Health Elements framework, this epidemiological shift reflects the convergence of multiple interacting domains: rapid urbanization (social) drove profound lifestyle transitions including reduced physical activity, dietary change, and increased metabolic risk (behavioral), producing a surge in diabetes and hypertension as primary drivers of kidney damage (biological) [46]. Concurrently, growing evidence links long-term exposure to fine particulate matter (PM2.5), heat stress, and climate-related environmental change to both indirect and direct nephrotoxic pathways, with effects that are further modified by behavioral factors—underscoring the cross-domain interaction between environmental and behavioral elements in shaping kidney health [46,47]. Digital health infrastructures—including large-scale electronic health record networks and emerging artificial intelligence (AI)-enabled tools such as electrocardiogram-based kidney function screening—now make it possible to investigate these cross-domain interactions at population scale and support early detection in primary care settings (technological) [48]. Taken together, this example illustrates how a modular Health Elements analysis is structured: each domain is activated by a specific hypothesized mechanism, interactions are framed as testable research questions, and the technological domain functions both as an explanatory element and as the infrastructure enabling population-scale investigation.
**Example 2: CKD of uncertain etiology (CKDu)** CKD of uncertain etiology (CKDu), observed among agricultural workers in Central America, South Asia, and Sri Lanka, exemplifies emergence—a health outcome that cannot be attributed to any single domain [49]. Genetic susceptibility to heat-induced kidney injury (biological) interacts with occupational exposure to extreme heat, dehydration, pesticides, and contaminated water sources (environmental and social), while behavioral adaptations such as reduced fluid intake during working hours further amplify risk [49,50]. In settings where digital infrastructure is absent or intermittent, limited surveillance capacity and low digital health literacy constrain both detection and etiological investigation—illustrating that the technological domain can be defined by its absence as much as its presence. Conversely, where digital infrastructure is available, technological elements may play a protective role: heat alert systems, wearable physiological monitoring, and telehealth-enabled occupational health services represent potential interventions at the intersection of environmental risk and behavioral response. CKDu thus illustrates how a modular analysis can be operationalized even when key domains are defined by absence rather than measurable presence, requiring proxy indicators, qualitative surveillance, and equity-conscious study designs.

## Operationalizing the Health Elements Framework

Translating Health Elements from a conceptual orientation into a scientifically tractable research framework requires careful attention to scope, measurement, modeling, and evaluation. The framework is not intended to encourage maximal data integration for its own sake. Rather, it provides a structure for identifying which elements and interactions are most relevant to particular health questions and decision contexts.

First, defining analytic scope is a foundational operational challenge. Health Elements explicitly acknowledge that not all domains are equally relevant for all outcomes. Attempting to operationalize the full framework simultaneously risks conceptual dilution and methodological instability. Instead, Health Elements support a modular approach, in which subsets of elements are selected based on hypothesized mechanisms, temporal considerations, and intended use. CKD serves as an instructive example for this modular approach: its pathophysiology involves sustained feedback loops across biological, behavioral, environmental, and social domains; its progression follows a long and measurable trajectory amenable to temporal modeling; and its early stages are frequently asymptomatic, with detection and clinical engagement shaped by health system access, individual awareness, and evolving digital health tools—making it a condition in which all 5 Health Elements domains are simultaneously operative and analytically tractable. These characteristics make CKD a well-suited context for illustrating how a modular Health Elements analysis is structured and operationalized across domains (Box 1). Explicit articulation of scope is therefore a prerequisite for meaningful operationalization.

Second, measurement across domains presents important and uneven challenges. Advances in biomedicine and digital health have substantially expanded the observability of biological and behavioral processes. Multiomics technologies allow detailed characterization of molecular pathways and immune states [20–22], while wearable devices and smartphones provide continuous measures of activity, sleep, and physiological signals. In contrast, social, environmental, and technological elements are often measured through proxies derived from surveys, administrative records, geospatial data, or platform metadata [26–29]. These measures vary widely in validity, resolution, and temporal alignment.

Uneven measurement across domains has important implications for integrated models. High-frequency biological or behavioral data may dominate inference simply because they are more granular, not because they are more causally influential. Conversely, distal but powerful social or environmental influences may be underweighted due to coarser measurement. Methodological research is therefore needed to assess how measurement error, missingness, and proxy validity propagate through multidomain models and how such imbalances can be mitigated. Sensitivity analyses, domain-specific weighting strategies, and explicit uncertainty quantification are essential components of responsible application.

Third, analytical methods must balance complexity with interpretability and causal relevance. A critical distinction is that “interaction” within the Health Elements framework does not refer to conventional statistical interaction terms, which estimate whether the marginal effect of one variable varies across levels of another within an additive or multiplicative model. Rather, we invoke feedback-producing, nonlinear, and context-dependent relationships among domains that evolve over time. In such systems, relationships are not merely effect modifiers but structurally interdependent processes in which outputs at one time point become inputs at subsequent stages.

This distinction has direct implications for method selection. System dynamics models capture feedback loops, time delays, and stock-and-flow structures, making them well suited for modeling how, for example, environmental exposures, behavioral adaptations, health system response, and technological change jointly shape disease trajectories [33,34]. Agent-based models simulate how individual-level interactions among biological, behavioral, social, environmental, and technological elements generate emergent population-level patterns that cannot be predicted from any single domain [35]. Network-based approaches represent the structure of cross-domain relationships and identify how perturbations propagate across elements [33]. These approaches differ from standard regression-based frameworks by treating interaction as dynamic structure rather than parametric modification.

These system-oriented approaches complement AI-based methods, particularly those designed for multimodal data, which offer powerful tools for modeling high-dimensional and nonlinear relationships [7–9,11]. However, predictive capacity along is insufficient. Integration with causal frameworks is necessary to distinguish structural feedback from correlation and to support inference about intervention effects.

A central methodological challenge is how to distinguish association from causation in integrated Health Elements models. Causal inference approaches, including target trial emulation, causal graphs, and counterfactual reasoning, provide tools for identifying modifiable elements and estimating intervention effects [36]. Hybrid approaches that combine AI algorithms with causal inference are increasingly explored to balance predictive accuracy with explanatory insight. Within the Health Elements framework, such approaches are particularly valuable for identifying interactions that are actionable rather than merely predictive.

Fourth, temporal dynamics require explicit methodological attention. Different domains operate at characteristically different time scales: biological processes occur over hours to days, behavioral patterns over weeks to months, and social and environmental conditions over years to decades. Cross-temporal interaction within the Health Elements framework refers to the process by which slowly changing conditions—such as socioeconomic deprivation or cumulative environmental exposure—modify the vulnerability within which faster-changing biological and behavioral processes operate; this is analytically distinct from contemporaneous statistical interaction. Appropriate methods include multiresolution time-series modeling that preserves each domain’s natural temporal resolution, lagged cross-domain interaction models with lag windows informed by prior literature, and life-course approaches that situate acute health events within cumulative exposure histories [37,38]. Sensitivity analyses over plausible lag specifications are essential. Longitudinal study designs with repeated measurements across domains are therefore central to meaningful operationalization.

Fifth, empirical evaluation and boundary setting are essential. The added complexity of Health Elements approaches must be justified empirically. Comparative evaluation against simpler models is essential to determine when integrated approaches provide meaningful gains in prediction, explanation, or equity. In some contexts, additional complexity may introduce noise or bias without improving insight. Identifying such boundary conditions is critical for preventing methodological inflation and for ensuring that Health Elements remain a disciplined scientific framework rather than an all-encompassing conceptual construct.

Finally, operationalization must remain sensitive to use context. Models intended for exploratory research, clinical decision support, public health surveillance, or policy analysis impose different requirements for interpretability, robustness, and timeliness. Health Elements do not specify a single analytic strategy; rather, they provide a conceptual structure within which analytic choices can be aligned with purpose and constraints.

## Implications for Public Health, Clinical Care, and Policy

The Health Elements framework has important implications for how public health systems, clinical practice, and policy making conceptualize and respond to health challenges in an increasingly complex and data-rich environment. In this framework, public health is not treated as an additional Health Element, but as the population-level system through which interactions among elements are analyzed and addressed. By emphasizing interactions among biological, behavioral, social, environmental, and technological domains, the framework encourages a shift away from siloed approaches toward more integrative and anticipatory strategies. This shift is particularly relevant as health systems confront multifactorial challenges—such as chronic disease, population aging, climate-related risks, and widening inequities—that cannot be effectively addressed through single-domain interventions.

In public health, Health Elements support a move from static risk factor surveillance toward dynamic, systems-oriented population health monitoring. Traditional public health indicators often rely on periodic surveys or aggregated metrics that provide limited insight into how risks evolve over time or interact across domains. An integrated Health Elements perspective enables public health researchers and practitioners to examine how behavioral patterns, social vulnerability, environmental exposures, and digital access jointly shape population health trajectories [28,29,31,39]. This approach is particularly relevant for preparedness and prevention, where early identification of interacting risks can inform timely and targeted responses. The framework also aligns with emerging approaches in precision public health, which seek to deliver the right intervention to the right population at the right time [11,40], while emphasizing that precision must be balanced with inclusivity. If certain populations remain underrepresented in digital data streams, data-intensive approaches risk reinforcing existing disparities.

For clinical care, Health Elements reinforce the importance of context-aware and person-centered medicine. While advances in precision medicine have enabled increasingly high-resolution biological stratification [20,21], clinical outcomes remain strongly shaped by behavioral adherence, social support, environmental exposures, and health system accessibility. Integrating these dimensions into clinical reasoning does not imply that clinicians must directly manage all domains. Rather, it highlights the value of situating biomedical information within a broader understanding of patients’ lived conditions. AI-enabled clinical decision support systems increasingly integrate multimodal data to inform diagnosis, risk stratification, and treatment planning [7–9]. Aligning such systems with Health Elements can improve relevance and robustness, but only if models are transparent, validated across diverse populations, and implemented in ways that support rather than override professional judgment. Without such safeguards, technological elements risk introducing new forms of opacity or bias into care delivery.

At the policy level, Health Elements offer a conceptual bridge between health and nonhealth sectors. Policies related to housing, transportation, environmental regulation, education, labor markets, and digital infrastructure all influence health, yet their effects are often assessed independently [28,29,41]. Framing these influences as interacting elements within a shared health system supports more coherent cross-sectoral policy analysis. It also provides a structure for examining trade-offs and synergies among interventions, helping policymakers anticipate unintended consequences and equity implications. Importantly, Health Elements do not provide specific policy solutions. Instead, they offer a systematic way to reason about how diverse policy domains contribute to health outcomes under different conditions, supporting more adaptive policymaking in which interventions are iteratively evaluated and refined as evidence and context evolve.

## Ethical, Equity, and Governance Considerations

The integration of digital technologies and AI into health research and decision-making raises profound ethical, equity, and governance challenges that must be addressed explicitly within the Health Elements framework. While integrated, data-driven approaches offer the potential to improve health understanding and outcomes, they also risk exacerbating existing inequities if their development and deployment are not guided by robust ethical principles.

Equity concerns arise from multiple sources. Access to digital technologies, connectivity, and data infrastructures varies widely across populations and regions. As a result, certain Health Elements—particularly behavioral or technological elements derived from digital traces—may be systematically underrepresented for disadvantaged groups [41]. This uneven visibility can bias analytic models and skew decisions toward populations with greater digital presence. By explicitly recognizing technological elements as determinants of health, the Health Elements framework brings these issues into analytic focus rather than treating them as external limitations.

Algorithmic bias also presents a related challenge. AI models trained on historical or incomplete data may reproduce or amplify structural inequities embedded in health systems and societies [42]. Within integrated Health Elements models, bias may arise not only from data within individual domains but also from how domains are weighted, represented, or combined. Rigorous auditing of multidomain models, including assessment of differential performance across population subgroups, is therefore essential.

Governance of data and AI systems is central to addressing these challenges. The linkage of multimodal data increases risks related to privacy, reidentification, and misuse, even when individual datasets are de-identified [43]. Ethical governance must address questions of data ownership, consent, security, and appropriate use, while balancing individual rights with potential collective benefits. Transparent documentation of data sources, modeling choices, and intended use is critical for accountability.

Participatory and inclusive governance approaches are particularly important when Health Elements analyses inform policy or resource allocation decisions that affect vulnerable populations. Engaging patients, communities, and other stakeholders in decisions about data use and AI deployment can help align technological innovation with societal values and lived experience. Such engagement also fosters trust, which is essential for the sustainability of digital health systems.

Ethical, equity, and governance considerations are not peripheral to the Health Elements framework but integral to its scientific and societal legitimacy. Without explicit attention to these issues, integrated approaches risk becoming instruments of technocratic optimization rather than tools for advancing equitable health.

## Future Research Directions

The Health Elements framework conceptualizes health as a system-level outcome shaped by interactions among biological, behavioral, social, environmental, and technological influences. Although this perspective reflects growing recognition of complexity in health, its value ultimately depends on whether it supports well-defined research questions, strengthens methodological practice, and generates insights beyond those produced by established determinant-based models. Several areas therefore require focused investigation.

A key issue concerns how health patterns arising from interacting influences can be defined and examined empirically. Many conventional epidemiological models rely on additive or linear assumptions, whereas Health Elements emphasize interaction, feedback, and nonlinear relationships. Future research should examine when models that explicitly represent interactions provide measurable advantages in explanation or prediction over simpler approaches and how such advantages can be demonstrated using transparent and reproducible criteria. An associated question is whether interaction-driven patterns can be distinguished empirically from the aggregated effects of individual elements.

Related challenges involve data representation and dimensionality. Health Elements encompass domains that differ substantially in scale, temporal stability, and measurability. A central research question is how to construct data representations that capture relevant cross-domain relationships while remaining analytically and computationally manageable. Addressing this issue will require systematic assessment of feature selection strategies, levels of aggregation, and dimensionality reduction methods, as well as careful evaluation of how representational choices influence inference, prediction, and equity across populations.

Measurement validity and bias are also central considerations. Although digital technologies have expanded data availability in some domains, coverage remains uneven across populations and contexts. Future studies should examine how missing data, proxy indicators, and measurement error affect integrated analyses and how these issues interact with existing social and structural inequalities. Increased data volume or resolution in selected domains should not be assumed to improve understanding if it displaces less detailed but more causally relevant information.

Methodological development is therefore an ongoing priority. Machine learning approaches offer useful tools for integrating heterogeneous data, but their application within Health Elements frameworks raises questions about interpretability, causal inference, and generalizability. Further work is needed to develop and evaluate hybrid approaches that combine machine learning with causal methods, particularly for identifying modifiable influences and estimating intervention effects. Robust evaluation across different populations and settings is essential to avoid context-specific conclusions. Within this methodological landscape, increasing attention has been given to large-scale AI models, including foundation models and large language models, which are trained on extensive and heterogeneous datasets [8,44]. While these models may facilitate flexible data integration, evidence of their added value in health research remains limited. Future research should assess whether they offer reproducible improvements over more targeted approaches and address challenges related to interpretability, data provenance, bias, and inappropriate generalization.

Temporal dynamics add further complexity. Interactions among Health Elements may change over time, with effects that accumulate, diminish, or shift across life stages or in response to changes in context. Longitudinal studies are therefore essential to examine how early-life configurations of Health Elements relate to later outcomes and how transitions, such as policy changes or technological developments, influence system behavior.

Finally, the Health Elements framework itself requires continued evaluation. Comparative studies should assess when integrated approaches offer meaningful advantages and when simpler models are sufficient. Attention to boundary conditions and negative findings will be important to avoid unnecessary complexity. In this sense, Health Elements should be understood as a research framework whose relevance depends on empirical performance and methodological discipline, rather than as a fixed or comprehensive model.

## Conclusion

Digital and AI-empowered Health Elements provide a structured approach for examining health within increasingly complex social, environmental, and technological contexts. By viewing health as the product of interactions among biological, behavioral, social, environmental, and technological domains, the framework extends determinant-based models and aligns health research with contemporary data ecosystems and analytical capabilities. Its contribution lies not in offering a comprehensive solution but in organizing evidence, analytical approaches, and ethical considerations in a way that supports systematic integration while guarding against unnecessary complexity. As health systems face growing chronic disease burdens, environmental and climate pressures, and rapid digital transformation, the Health Elements framework may help enable more adaptive, equitable, and evidence-informed approaches to health research, practice, and policy.

## References

[1] World Health Organization. Constitution of the World Health Organization. WHO Chron. 1947;1-2:29–41. 20267861

[2] Whitmee S, Haines A, Beyrer C, Boltz F, Capon AG, de Souza Dias BF, Ezeh A, Frumkin H, Gong P, Head P, et al. Safeguarding human health in the Anthropocene epoch: Report of the Rockefeller Foundation–Lancet Commission on planetary health. Lancet. 2015;386(10007):1973–2028. 26188744 10.1016/S0140-6736(15)60901-1

[3] Marmot M, Allen J, Bell R, Bloomer E, Goldblatt P. WHO European review of social determinants of health and the health divide. Lancet. 2012;380(9846):1011–1029. 22964159 10.1016/S0140-6736(12)61228-8

[4] GBD 2019 Risk Factors Collaborators. Global burden of 87 risk factors in 204 countries and territories, 1990-2019: A systematic analysis for the Global Burden of Disease Study 2019. Lancet. 2020;396(10258):1223–1249. 33069327 10.1016/S0140-6736(20)30752-2 PMC7566194

[5] Bambra C, Riordan R, Ford J, Matthews F. The COVID-19 pandemic and health inequalities. J Epidemiol Community Health. 2020;74(11):964–968. 32535550 10.1136/jech-2020-214401 PMC7298201

[6] Diez Roux AV. Complex systems thinking and current impasses in health disparities research. Am J Public Health. 2011;101(9):1627–1634. 21778505 10.2105/AJPH.2011.300149 PMC3154209

[7] Rajkomar A, Dean J, Kohane I. Machine learning in medicine. N Engl J Med. 2019;380(14):1347–1358. 30943338 10.1056/NEJMra1814259

[8] Topol EJ. High-performance medicine: The convergence of human and artificial intelligence. Nat Med. 2019;25(1):44–56. 30617339 10.1038/s41591-018-0300-7

[9] Esteva A, Robicquet A, Ramsundar B, Kuleshov V, De Pristo M, Chou K, Cui C, Corrado G, Thrun S, Dean J. A guide to deep learning in healthcare. Nat Med. 2019;25(1):24–29. 30617335 10.1038/s41591-018-0316-z

[10] Beam AL, Kohane IS. Big data and machine learning in health care. JAMA. 2018;319(13):1317–1318. 29532063 10.1001/jama.2017.18391

[11] Roberts MC, Holt KE, Del Fiol G, Baccarelli AA, Allen CG. Precision public health in the era of genomics and big data. Nat Med. 2024;30(7):1865–1873. 38992127 10.1038/s41591-024-03098-0 PMC12017803

[12] Marmot M, Wilkinson R. *Social determinants of health*. Oxford: Oxford University Press; 2006.

[13] Galea S, Riddle M, Kaplan GA. Causal thinking and complex system approaches in epidemiology. Int J Epidemiol. 2010;39(1):97–106. 19820105 10.1093/ije/dyp296 PMC2912489

[14] Richardson S, Lawrence K, Schoenthaler AM, Mann D. A framework for digital health equity. NPJ Digit Med. 2022;5(1):119. 35982146 10.1038/s41746-022-00663-0 PMC9387425

[15] Kickbusch I, Piselli D, Agrawal A, Balicer R, Banner O, Adelhardt M, Capobianco E, Fabian C, Singh Gill A, Lupton D, et al. The lancet and financial times commission on governing health futures 2030: Growing up in a digital world. Lancet. 2021;398(10312):1727–1776. 34706260 10.1016/S0140-6736(21)01824-9

[16] Chidambaram S, Jain B, Jain U, Mwavu R, Baru R, Thomas B, Greaves F, Jayakumar S, Jain P, Rojo M, et al. An introduction to digital determinants of health. PLOS Digit Health. 2024;3(1): Article e0000346. 38175828 10.1371/journal.pdig.0000346 PMC10766177

[17] Crawford A, Serhal E. Digital health equity and COVID-19: The innovation curve cannot reinforce the social gradient of health. J Med Internet Res. 2020;22(6): Article e19361. 32452816 10.2196/19361 PMC7268667

[18] Obermeyer Z, Emanuel EJ. Predicting the future—Big data, machine learning, and clinical medicine. N Engl J Med. 2016;375(13):1216. 27682033 10.1056/NEJMp1606181 PMC5070532

[19] DHIT. DHIT Periodic Table Whitepaper. [accessed 1 Mar 2026 2026] https://dhitglobal.org/wp-content/uploads/2025/09/DHIT-Periodic-Table-Whitepaper-090325-03-2.pdf

[20] Collins FS, Varmus H. A new initiative on precision medicine. N Engl J Med. 2015;372(9):793–795. 25635347 10.1056/NEJMp1500523 PMC5101938

[21] Manolio TA, Chisholm RL, Ozenberger B, Roden DM, Williams MS, Wilson R, Bick D, Bottinger EP, Brilliant MH, Eng C, et al. Implementing genomic medicine in the clinic: The future is here. Genet Med. 2013;15(4):258–267. 23306799 10.1038/gim.2012.157 PMC3835144

[22] López-Otín C, Blasco MA, Partridge L, Serrano M, Kroemer G. Hallmarks of aging: An expanding universe. Cell. 2023;186(2):243–278. 36599349 10.1016/j.cell.2022.11.001

[23] Hood L, Friend SH. Predictive, personalized, preventive, participatory(P4) cancer medicine. Nat Rev Clin Oncol. 2011;8(3):184–187. 21364692 10.1038/nrclinonc.2010.227

[24] Doherty A, Jackson D, Hammerla N, Plötz T, Olivier P, Granat MH, White T, van Hees VT, Trenell MI, Owen CG, et al. Large scale population assessment of physical activity using wrist worn accelerometers: The UK biobank study. PLoS One. 2017;12(2): Article e0169649. 28146576 10.1371/journal.pone.0169649 PMC5287488

[25] Sameh A, Rostami M, Oussalah M, Korpelainen R, Farrahi V. Digital phenotypes and digital biomarkers for health and diseases: A systematic review of machine learning approaches utilizing passive non-invasive signals collected via wearable devices and smartphones. Artif Intell Rev. 2024;58(2):66.

[26] Christakis NA, Fowler JH. The spread of obesity in a large social network over 32 years. N Engl J Med. 2007;357(4):370–379. 17652652 10.1056/NEJMsa066082

[27] Guo C, Yu T, Bo Y, Lin C, Chang L-Y, Wong Martin CS, Yu Z, AKH L, Tam T, Lao XQ. Long-term exposure to fine particulate matter and mortality a longitudinal cohort study of 400,459 adults. Epidemiology. 2022;33(3):309–317. 35067568 10.1097/EDE.0000000000001464

[28] Giles-Corti B, Vernez-Moudon A, Reis R, Turrell G, Dannenberg AL, Badland H, Foster S, Lowe M, Sallis JF, Stevenson M, et al. City planning and population health: A global challenge. Lancet. 2016;388(10062):2912–2924. 27671668 10.1016/S0140-6736(16)30066-6

[29] Nieuwenhuijsen MJ. Urban and transport planning, environmental exposures and health-new concepts, methods and tools to improve health in cities. Environ Health. 2016;15(Suppl 1):38. 26960529 10.1186/s12940-016-0108-1 PMC4895603

[30] Wakeman M, Buckman DW, El-Toukhy S. Disparities in digital health care use in 2022. JAMA Netw Open. 2025;8(4): Article e255359. 40244585 10.1001/jamanetworkopen.2025.5359 PMC12550827

[31] Arias López MP, Ong BA, Borrat Frigola X, Fernández AL, Hicklent RS, AJT O, Rocimo AM, Celi LA, et al. Digital literacy as a new determinant of health: A scoping review. PLOS Digit Health. 2023;2(10): Article e0000279. 37824584 10.1371/journal.pdig.0000279 PMC10569540

[32] Lehne M, Sass J, Essenwanger A, Schepers J, Thun S. Why digital medicine depends on interoperability. NPJ Digit Med. 2019;2(1):79. 31453374 10.1038/s41746-019-0158-1 PMC6702215

[33] Luke DA, Stamatakis KA. Systems science methods in public health: Dynamics, networks, and agents. Annu Rev Public Health. 2012;33:357–376. 22224885 10.1146/annurev-publhealth-031210-101222 PMC3644212

[34] Li Y, Kong N, Lawley M, Weiss L, Pagan JA. Advancing the use of evidence-based decision-making in local health departments with systems science methodologies. Am J Public Health. 2015;105 Suppl 2(Suppl 2):S217–S222. 25689181 10.2105/AJPH.2014.302077 PMC4355700

[35] Li Y, Lawley MA, Siscovick DS, Zhang D, Pagan JA. Agent-based modeling of chronic diseases: A narrative review and future research directions. Prev Chronic Dis. 2016;13:E69. 27236380 10.5888/pcd13.150561 PMC4885681

[36] Hernán MA, Robins JM. Using big data to emulate a target trial when a randomized trial is not available. Am J Epidemiol. 2016;183(8):758–764. 26994063 10.1093/aje/kwv254 PMC4832051

[37] Wagner C, Carmeli C, Jackisch J, Kivimäki M, van der Linden BWA, Cullati S, Chiolero A. Life course epidemiology and public health. Lancet Public Health. 2024;9(4):e261–e269. 38553145 10.1016/S2468-2667(24)00018-5

[38] Lynch J, Smith GD. A life course approach to chronic disease epidemiology. Annu Rev Public Health. 2005;26(1):1–35. 15760279 10.1146/annurev.publhealth.26.021304.144505

[39] Thomson MC, Stanberry LR. Climate change and vectorborne diseases. N Engl J Med. 2022;387(21):1969–1978. 36416768 10.1056/NEJMra2200092

[40] Khoury MJ, Iademarco MF, Riley WT. Precision public health for the era of precision medicine. Am J Prev Med. 2015;50(3):398. 26547538 10.1016/j.amepre.2015.08.031 PMC4915347

[41] Van Dijk JA. Digital divide research, achievements and shortcomings. Poetics. 2006;34(4-5):221–235.

[42] Obermeyer Z, Powers B, Vogeli C, Mullainathan S. Dissecting racial bias in an algorithm used to manage the health of populations. Science. 2019;366(6464):447–453. 31649194 10.1126/science.aax2342

[43] Price WN, Cohen IG. Privacy in the age of medical big data. Nat Med. 2019;25(1):37–43. 30617331 10.1038/s41591-018-0272-7 PMC6376961

[44] Singhal K, Azizi S, Tu T, et al. Large language models encode clinical knowledge. Nature. 2023;620(7972):172–180. 37438534 10.1038/s41586-023-06291-2 PMC10396962

[45] Zhang L, Long J, Jiang W, et al. Trends in chronic kidney disease in China. N Engl J Med. 2016;375(9):905–906. 27579659 10.1056/NEJMc1602469

[46] Zhang L, Santos ACS Jr, Anandh U, Saran R, Zhao MH. Effects of social determinants of health on the landscape of kidney disease. Nat Rev Nephrol. 2026. 10.1038/s41581-026-01052-6 41578009

[47] Wang W, Zhang X, Zhang M, et al. Extreme temperature events, “Life’s Essential 8”, and prevalence of chronic kidney disease: A nationally representative surveillance in China. Environ Int. 2024;194: Article 109176. 39657396 10.1016/j.envint.2024.109176

[48] Li J, Aguirre AD, Moura V, Jin J, Liu C, Zhong L, Sun C, Clifford G, Westover MB, Hong S. An electrocardiogram foundation model built on over 10 million recordings. NEJM AI. 2025;2(7): Article 10.1056/aioa2401033. 10.1056/aioa2401033 PMC12327759 40771651

[49] Diwyanjalee GR, Wimalawansa SJ. The silent epidemic of CKDu: Advances, challenges, and strategies for sustainable eradication-a narrative review. Clin Exp Nephrol. 2025;29(10):1313–1330. 40974452 10.1007/s10157-025-02751-3

[50] Friedman DJ, Leone DA, Amador JJ, Kupferman J, Francey LJ, Lopez-Pilarte D, Lau J, Delgado I, Yih WK, Salinas A, et al. Genetic risk factors for Mesoamerican nephropathy. Proc Natl Acad Sci USA. 2024;121(49): Article e2404848121. 39585978 10.1073/pnas.2404848121 PMC11626114

